# Supplementary material for: Fitness costs of female choosiness are low in a socially monogamous songbird
Source: PLoS Biol. 2021 Nov 4;19(11):e3001257. doi: 10.1371/journal.pbio.3001257 (PMC8568113; doi:10.1371/journal.pbio.3001257)
Supplement: S1 Fig — (DOCX) [file pbio.3001257.s001.docx]

**S1 Fig. Kaplan-Meier plot showing the time taken to social pairing under the two experimental treatments.** Solid lines indicate the proportion of birds that are still unpaired over the course of the experiment. Dashed lines indicate 95% CIs (generated using the survfit function of the survminer package V0.4.9 [1]. The data underlying this Figure can be found in <https://osf.io/6e8np/>.


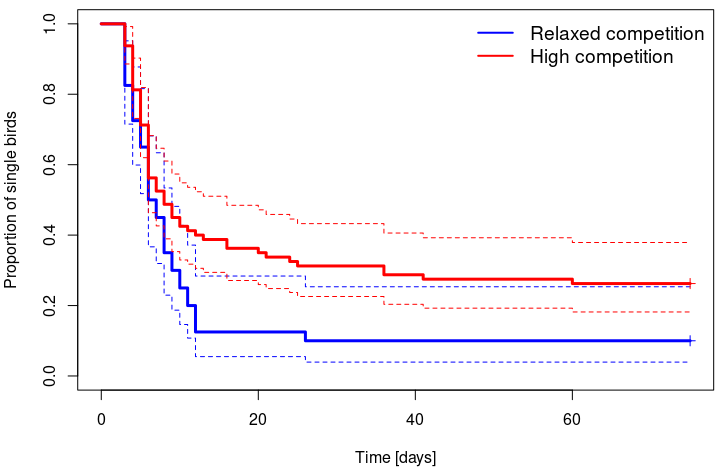


1. Alboukadel K, Marcin K, Przemyslaw B. survminer: Drawing Survival Curves using 'ggplot2'. 0.4.9 ed2021.
