# Supplementary material for: Fitness costs of female choosiness are low in a socially monogamous songbird
Source: PLoS Biol. 2021 Nov 4;19(11):e3001257. doi: 10.1371/journal.pbio.3001257 (PMC8568113; doi:10.1371/journal.pbio.3001257)
Supplement: S1 Table — (DOCX) [file pbio.3001257.s002.docx]

**S1 Table. Female relative fitness as a function of treatment and confounding factors.**

| Model 1 |  | Estimate | SE | *t* | *p* |
| --- | --- | --- | --- | --- | --- |
| Model 1a |  |  |  |  |  |
| Intercept |  | 0.955 | 0.097 |  |  |
| Treatment (high competition) |  | 0.067 | 0.119 | 0.57 | 0.57 |
| Inbreeding coefficient (centred) |  | -3.143 | 1.126 | -2.79 | 0.006 |
|  |  |  |  |  |  |
| Model 1b |  |  |  |  |  |
| Intercept |  | 0.914 | 0.102 |  |  |
| Treatment (high competition) |  | 0.128 | 0.128 | 1.01 | 0.32 |
| Inbreeding coefficient (centred) |  | -3.535 | 1.256 | -2.82 | 0.006 |
| Number of peers in natal aviary (centred) |  | -0.017 | 0.012 | -1.46 | 0.15 |
| Relative fitness of mother (centred) |  | 0.034 | 0.020 | 1.66 | 0.099 |
|  |  |  |  |  |  |
