# Supplementary material for: Fitness costs of female choosiness are low in a socially monogamous songbird
Source: PLoS Biol. 2021 Nov 4;19(11):e3001257. doi: 10.1371/journal.pbio.3001257 (PMC8568113; doi:10.1371/journal.pbio.3001257)
Supplement: S2 Table — (DOCX) [file pbio.3001257.s003.docx]

**S2 Table. Number of genetically verified eggs laid per female as a function of treatment and female inbreeding coefficient.**

| Model 2 | Levels | Estimate | SE | df | *t* | *p* |
| --- | --- | --- | --- | --- | --- | --- |
| Random effects (variance) |  |  |  |  |  |  |
| Natal aviary | 15 | 0.16 |  |  |  |  |
| Experimental aviary | 10 | 0.22 |  |  |  |  |
| Residual | 120 | 12.46 |  |  |  |  |
|  |  |  |  |  |  |  |
| Fixed effects |  |  |  |  |  |  |
| Intercept |  | 8.56 | 0.59 | 34.8 |  |  |
| Treatment (high competition) |  | 0.64 | 0.70 | 20.1 | 0.91 | 0.37 |
| Inbreeding coefficient (centred) |  | -18.83 | 6.73 | 79.1 | -2.80 | 0.0065 |
|  |  |  |  |  |  |  |
