# Supplementary material for: Fitness costs of female choosiness are low in a socially monogamous songbird
Source: PLoS Biol. 2021 Nov 4;19(11):e3001257. doi: 10.1371/journal.pbio.3001257 (PMC8568113; doi:10.1371/journal.pbio.3001257)
Supplement: S3 Table — (DOCX) [file pbio.3001257.s004.docx]

**S3 Table. Latency (in days, log10-transformed) to lay the first genetically verified egg as a function of treatment and female inbreeding coefficient.** Note that three out of 120 females did not lay eggs and were assigned a latency of 75 days (end of experiment).

| Model 3 | Levels | Estimate | SE | df | *t* | *p* |
| --- | --- | --- | --- | --- | --- | --- |
| Random effects (variance) |  |  |  |  |  |  |
| Natal aviary | 15 | 0.0061 |  |  |  |  |
| Experimental aviary | 10 | 0 |  |  |  |  |
| Residual | 120 | 0.1012 |  |  |  |  |
|  |  |  |  |  |  |  |
| Fixed effects |  |  |  |  |  |  |
| Intercept |  | 0.92 | 0.057 | 37.5 |  |  |
| Treatment (high competition) |  | -0.033 | 0.069 | 45.8 | -0.48 | 0.63 |
| Inbreeding coefficient (centred) |  | 1.07 | 0.623 | 95.1 | 1.72 | 0.09 |
|  |  |  |  |  |  |  |
