# Supplementary material for: Fitness costs of female choosiness are low in a socially monogamous songbird
Source: PLoS Biol. 2021 Nov 4;19(11):e3001257. doi: 10.1371/journal.pbio.3001257 (PMC8568113; doi:10.1371/journal.pbio.3001257)
Supplement: S4 Table — (DOCX) [file pbio.3001257.s005.docx]

**S4 Table. Probability of remaining socially unpaired (not recorded participating in one of 106 nest-attending pairs) as a function of treatment and female inbreeding coefficient (binomial model on n=120 females).**

| Model 4 | Levels | Estimate | SE | *z* | *p* |
| --- | --- | --- | --- | --- | --- |
| Random effects (variance) |  |  |  |  |  |
| Natal aviary | 15 | 0.04 |  |  |  |
| Experimental aviary | 10 | 0.32 |  |  |  |
|  |  |  |  |  |  |
| Fixed effects |  |  |  |  |  |
| Intercept |  | -2.63 | 0.66 |  |  |
| Treatment (high competition) |  | 1.49 | 0.71 | 2.11 | 0.035* |
| Inbreeding coefficient (centred) |  | 20.4 | 7.00 | 2.91 | 0.0036 |
|  |  |  |  |  |  |

* Note that this p-value is calculated from the z-value assuming infinite df. A more conservative p-value assuming 9df is p = 0.064 (as shown in Table 1).
