# Supplementary material for: Fitness costs of female choosiness are low in a socially monogamous songbird
Source: PLoS Biol. 2021 Nov 4;19(11):e3001257. doi: 10.1371/journal.pbio.3001257 (PMC8568113; doi:10.1371/journal.pbio.3001257)
Supplement: S5 Table — (DOCX) [file pbio.3001257.s006.docx]

**S5 Table. Number of social pair bonds observed per female (range 0-2) as a function of treatment and female inbreeding coefficient (Gaussian mixed-effect model).**

| Model 5 | Levels | Estimate | SE | df | *t* | *p* |
| --- | --- | --- | --- | --- | --- | --- |
| Random effects (variance) |  |  |  |  |  |  |
| Natal aviary | 15 | 0.015 |  |  |  |  |
| Experimental aviary | 10 | 0.007 |  |  |  |  |
| Residual | 120 | 0.248 |  |  |  |  |
|  |  |  |  |  |  |  |
| Fixed effects |  |  |  |  |  |  |
| Intercept |  | 0.931 | 0.092 | 30.8 |  |  |
| Treatment (high competition) |  | -0.088 | 0.108 | 28.8 | -0.82 | 0.42 |
| Inbreeding coefficient (centred) |  | -3.18 | 0.995 | 96.7 | -3.20 | 0.0019 |
|  |  |  |  |  |  |  |
