# Supplementary material for: Fitness costs of female choosiness are low in a socially monogamous songbird
Source: PLoS Biol. 2021 Nov 4;19(11):e3001257. doi: 10.1371/journal.pbio.3001257 (PMC8568113; doi:10.1371/journal.pbio.3001257)
Supplement: S6 Table — (DOCX) [file pbio.3001257.s007.docx]

**S6 Table. Number of assortative social pair bonds observed per female (range 0-2) as a function of treatment and female inbreeding coefficient (Gaussian mixed-effect model).**

| Model 6 | Levels | Estimate | SE | df | *t* | *p* |
| --- | --- | --- | --- | --- | --- | --- |
| Random effects (variance) |  |  |  |  |  |  |
| Natal aviary | 15 | 0 |  |  |  |  |
| Experimental aviary | 10 | 0 |  |  |  |  |
| Residual | 120 | 0.221 |  |  |  |  |
|  |  |  |  |  |  |  |
| Fixed effects |  |  |  |  |  |  |
| Intercept |  | 0.90 | 0.074 | 117 |  |  |
| Treatment (high competition) |  | -0.45 | 0.091 | 117 | -4.96 | <0.0001 |
| Inbreeding coefficient (centred) |  | -2.26 | 0.863 | 117 | -2.62 | 0.0099 |
|  |  |  |  |  |  |  |
