# Supplementary material for: Fitness costs of female choosiness are low in a socially monogamous songbird
Source: PLoS Biol. 2021 Nov 4;19(11):e3001257. doi: 10.1371/journal.pbio.3001257 (PMC8568113; doi:10.1371/journal.pbio.3001257)
Supplement: S7 Table — (DOCX) [file pbio.3001257.s008.docx]

**S7 Table. Number of disassortative social pair bonds observed per female (range 0-2) as a function of treatment and female inbreeding coefficient (Gaussian mixed-effect model).**

| Model 7 | Levels | Estimate | SE | df | *t* | *p* |
| --- | --- | --- | --- | --- | --- | --- |
| Random effects (variance) |  |  |  |  |  |  |
| Natal aviary | 15 | 0.011 |  |  |  |  |
| Experimental aviary | 10 | 0.011 |  |  |  |  |
| Residual | 120 | 0.221 |  |  |  |  |
|  |  |  |  |  |  |  |
| Fixed effects |  |  |  |  |  |  |
| Intercept |  | 0.035 | 0.088 | 29.2 |  |  |
| Treatment (high competition) |  | 0.365 | 0.100 | 21.8 | 3.65 | 0.0014 |
| Inbreeding coefficient (centred) |  | -0.824 | 0.944 | 97.6 | -0.87 | 0.38 |
|  |  |  |  |  |  |  |
