# Supplementary material for: Fitness costs of female choosiness are low in a socially monogamous songbird
Source: PLoS Biol. 2021 Nov 4;19(11):e3001257. doi: 10.1371/journal.pbio.3001257 (PMC8568113; doi:10.1371/journal.pbio.3001257)
Supplement: S8 Table — (DOCX) [file pbio.3001257.s009.docx]

**S8 Table. Latency (in days, log10-transformed) to the first recorded egg in a clutch attended as one of the 106 social pairs as a function of treatment and female inbreeding coefficient.** Note that 25 out of 120 females (21%) did not participate in any of these pair bonds and were assigned a latency of 75 days (end of experiment). For an alternative analysis based on a Cox proportional hazard model see S17 Table.

| Model 8 | Levels | Estimate | SE | df | *t* | *p* |
| --- | --- | --- | --- | --- | --- | --- |
| Random effects (variance) |  |  |  |  |  |  |
| Natal aviary | 15 | 0 |  |  |  |  |
| Experimental aviary | 10 | 0.006 |  |  |  |  |
| Residual | 120 | 0.200 |  |  |  |  |
|  |  |  |  |  |  |  |
| Fixed effects |  |  |  |  |  |  |
| Intercept |  | 0.897 | 0.075 | 38.2 |  |  |
| Treatment (high competition) |  | 0.234 | 0.087 | 105.9 | 2.70 | 0.008 |
| Inbreeding coefficient (centred) |  | 3.650 | 0.852 | 88.6 | 4.28 | 0.00005 |
|  |  |  |  |  |  |  |
