# Supplementary material for: Fitness costs of female choosiness are low in a socially monogamous songbird
Source: PLoS Biol. 2021 Nov 4;19(11):e3001257. doi: 10.1371/journal.pbio.3001257 (PMC8568113; doi:10.1371/journal.pbio.3001257)
Supplement: S9 Table — (DOCX) [file pbio.3001257.s010.docx]

**S9 Table. Number of clutches attended as a single mother (range 0-2) as a function of treatment and female inbreeding coefficient (Gaussian mixed-effect model).**

| Model 9 | Levels | Estimate | SE | df | *t* | *p* |
| --- | --- | --- | --- | --- | --- | --- |
| Random effects (variance) |  |  |  |  |  |  |
| Natal aviary | 15 | 0.009 |  |  |  |  |
| Experimental aviary | 10 | 0 |  |  |  |  |
| Residual | 120 | 0.155 |  |  |  |  |
|  |  |  |  |  |  |  |
| Fixed effects |  |  |  |  |  |  |
| Intercept |  | 0.127 | 0.070 | 33.3 |  |  |
| Treatment (high competition) |  | 0.049 | 0.085 | 40.8 | 0.58 | 0.57 |
| Inbreeding coefficient (centred) |  | 1.092 | 0.768 | 90.7 | 1.42 | 0.16 |
|  |  |  |  |  |  |  |
