# Supplementary material for: Fitness costs of female choosiness are low in a socially monogamous songbird
Source: PLoS Biol. 2021 Nov 4;19(11):e3001257. doi: 10.1371/journal.pbio.3001257 (PMC8568113; doi:10.1371/journal.pbio.3001257)
Supplement: S10 Table — (DOCX) [file pbio.3001257.s011.docx]

**S10 Table. Number of eggs which the female took care of (recorded as a social mother in any pairing constellation) as a function of treatment and female inbreeding coefficient.**

| Model 10 | Levels | Estimate | SE | df | *t* | *p* |
| --- | --- | --- | --- | --- | --- | --- |
| Random effects (variance) |  |  |  |  |  |  |
| Natal aviary | 15 | 0 |  |  |  |  |
| Experimental aviary | 10 | 1.13 |  |  |  |  |
| Residual | 120 | 10.14 |  |  |  |  |
|  |  |  |  |  |  |  |
| Fixed effects |  |  |  |  |  |  |
| Intercept |  | 7.41 | 0.61 | 28.7 |  |  |
| Treatment (high competition) |  | -0.78 | 0.62 | 108.1 | -1.27 | 0.21 |
| Inbreeding coefficient (centred) |  | -23.65 | 6.34 | 111.7 | -3.73 | 0.0003 |
|  |  |  |  |  |  |  |
