# Supplementary material for: Fitness costs of female choosiness are low in a socially monogamous songbird
Source: PLoS Biol. 2021 Nov 4;19(11):e3001257. doi: 10.1371/journal.pbio.3001257 (PMC8568113; doi:10.1371/journal.pbio.3001257)
Supplement: S11 Table — (DOCX) [file pbio.3001257.s012.docx]

**S11 Table. Number of genetically verified eggs by a female that were cared for by another female as a function of treatment and female inbreeding coefficient.**

| Model 11 | Levels | Estimate | SE | df | *t* | *p* |
| --- | --- | --- | --- | --- | --- | --- |
| Random effects (variance) |  |  |  |  |  |  |
| Natal aviary | 15 | 0 |  |  |  |  |
| Experimental aviary | 10 | 0 |  |  |  |  |
| Residual | 120 | 4.15 |  |  |  |  |
|  |  |  |  |  |  |  |
| Fixed effects |  |  |  |  |  |  |
| Intercept |  | 0.80 | 0.32 | 117 |  |  |
| Treatment (high competition) |  | 0.83 | 0.39 | 117 | 2.10 | 0.038 |
| Inbreeding coefficient (centred) |  | 2.70 | 3.74 | 117 | 0.72 | 0.47 |
|  |  |  |  |  |  |  |
