# Supplementary material for: Fitness costs of female choosiness are low in a socially monogamous songbird
Source: PLoS Biol. 2021 Nov 4;19(11):e3001257. doi: 10.1371/journal.pbio.3001257 (PMC8568113; doi:10.1371/journal.pbio.3001257)
Supplement: S12 Table — (DOCX) [file pbio.3001257.s013.docx]

**S12 Table. Number of genetically verified eggs per female that she did not take care of as a function of treatment and female inbreeding coefficient.**

| Model 12 | Levels | Estimate | SE | df | *t* | *p* |
| --- | --- | --- | --- | --- | --- | --- |
| Random effects (variance) |  |  |  |  |  |  |
| Natal aviary | 15 | 0.23 |  |  |  |  |
| Experimental aviary | 10 | 0 |  |  |  |  |
| Residual | 120 | 6.92 |  |  |  |  |
|  |  |  |  |  |  |  |
| Fixed effects |  |  |  |  |  |  |
| Intercept |  | 1.11 | 0.45 | 39.0 |  |  |
| Treatment (high competition) |  | 1.48 | 0.55 | 44.0 | 2.72 | 0.0094 |
| Inbreeding coefficient (centred) |  | 3.51 | 5.03 | 86.5 | 0.70 | 0.49 |
|  |  |  |  |  |  |  |
