# Supplementary material for: Fitness costs of female choosiness are low in a socially monogamous songbird
Source: PLoS Biol. 2021 Nov 4;19(11):e3001257. doi: 10.1371/journal.pbio.3001257 (PMC8568113; doi:10.1371/journal.pbio.3001257)
Supplement: S13 Table — (DOCX) [file pbio.3001257.s014.docx]

**S13 Table. Relative counts of eggs that the female dumped (in a strict sense) versus her remaining eggs as a function of treatment and female inbreeding coefficient (binomial mixed-effect model).**

| Model 13 | Levels | Estimate | SE | *z* | *p* |
| --- | --- | --- | --- | --- | --- |
| Random effects (variance) |  |  |  |  |  |
| Female identity | 120 | 1.97 |  |  |  |
| Natal aviary | 15 | 0 |  |  |  |
| Experimental aviary | 10 | 0 |  |  |  |
|  |  |  |  |  |  |
| Fixed effects |  |  |  |  |  |
| Intercept |  | -2.96 | 0.35 |  |  |
| Treatment (high competition) |  | 0.87 | 0.39 | 2.21 | 0.027* |
| Inbreeding coefficient (centred) |  | 6.76 | 3.75 | 1.80 | 0.071 |
|  |  |  |  |  |  |

* Note that this p-value is calculated from the z-value assuming infinite df. A more conservative p-value assuming 113df is p = 0.029 (as shown in Table 1).
