# Supplementary material for: Fitness costs of female choosiness are low in a socially monogamous songbird
Source: PLoS Biol. 2021 Nov 4;19(11):e3001257. doi: 10.1371/journal.pbio.3001257 (PMC8568113; doi:10.1371/journal.pbio.3001257)
Supplement: S15 Table — (DOCX) [file pbio.3001257.s016.docx]

**S15 Table. Relative counts of eggs that developed into independent young and her remaining eggs that did not reach independence as a function of treatment and female inbreeding coefficient (binomial mixed-effect model).**

| Model 15 | Levels | Estimate | SE | *z* | *p* |
| --- | --- | --- | --- | --- | --- |
| Random effects (variance) |  |  |  |  |  |
| Female identity | 120 | 0.78 |  |  |  |
| Natal aviary | 15 | 0 |  |  |  |
| Experimental aviary | 10 | 0.08 |  |  |  |
|  |  |  |  |  |  |
| Fixed effects |  |  |  |  |  |
| Intercept |  | 0.064 | 0.21 |  |  |
| Treatment (high competition) |  | -0.036 | 0.23 | -0.16 | 0.87* |
| Inbreeding coefficient (centred) |  | -6.51 | 2.85 | -2.29 | 0.022 |
|  |  |  |  |  |  |

* Note that this p-value is calculated from the z-value assuming infinite df. A more conservative p-value assuming 40df is p = 0.88 (as shown in Table 1).
