# Supplementary material for: Fitness costs of female choosiness are low in a socially monogamous songbird
Source: PLoS Biol. 2021 Nov 4;19(11):e3001257. doi: 10.1371/journal.pbio.3001257 (PMC8568113; doi:10.1371/journal.pbio.3001257)
Supplement: S16 Table — (DOCX) [file pbio.3001257.s017.docx]

**S16 Table. Descriptive statistics on extrapair mating by three groups of females (dependent on competition treatment and social pairing status).**

| Dependent variable | Low  competition  paired assortatively | High competition paired assortatively | High competition paired disassortatively |
| --- | --- | --- | --- |
| % females with EPP (N with EPP / N total females) | 66 (23/35) | 44 (12/27) | 81 (17/21) |
| % EPP (of eggs) in females with EPP ± SE | 55 ± 7 | 41 ± 8 | 62 ± 9 |
| % EPP of all eggs (N EPP / N total eggs) | 36 (112/312) | 18 (46/252) | 44 (81/183) |
| % assortative of EP-eggs (N assortative / N EPP) | 89 (100/112) | 65 (30/46) | 70 (57/81) |
| % assortative of EP-relationships (N assortative / N total EP-relationships) | 87 (40/46) | 53 (9/17) | 59 (19/32) |
| % assortative expected of EP-relationships (N assortative / N total EP-males in aviary) | 64 (7/11) | 27 (3/11) | 36 (4/11) |
| N eggs sired by assortative EP-male per relationship ± SE (N relationships) | 2.5 ± 0.3 (40) | 3.3 ± 0.6 (9) | 3.0 ± 0.5 (19) |
| N eggs sired by disassortative EP-male per relationship ± SE (N relationships) | 2.0 ± 0.4 (6) | 2.0 ± 0.3 (8) | 1.8 ± 0.3 (13) |

**EPP**: extra-pair paternity

**EP**: extra-pair
