# Supplementary material for: Fitness costs of female choosiness are low in a socially monogamous songbird
Source: PLoS Biol. 2021 Nov 4;19(11):e3001257. doi: 10.1371/journal.pbio.3001257 (PMC8568113; doi:10.1371/journal.pbio.3001257)
Supplement: S17 Table — (DOCX) [file pbio.3001257.s018.docx]

**S17 Table. Cox proportional hazard model on the probability of social pairing over time (i.e. time to the first recorded egg in a clutch attended as one of the 106 social pairs) as a function of treatment and female inbreeding coefficient.** Note that 25 out of 120 females (21%) did not participate in any of these pair bonds until the end of the experiment (75 days), hence there are 95 recorded events of pairing. See also S1 Figure.

| Model 8a | Levels | Estimate | Exp of Estimate | SE of Estimate | *z* | *p* |
| --- | --- | --- | --- | --- | --- | --- |
| Random effects (variance) |  |  |  |  |  |  |
| Natal aviary | 15 | 0.0004 |  |  |  |  |
| Experimental aviary | 10 | 0.033 |  |  |  |  |
|  |  |  |  |  |  |  |
| Fixed effects |  |  |  |  |  |  |
| Treatment (high competition) |  | -0.482 | 0.618* | 0.216 | -2.24 | 0.025 |
| Inbreeding coefficient (centred) |  | -8.95 | 0.0001 | 2.83 | -3.16 | 0.0016 |
|  |  |  |  |  |  |  |

* 95% CI of the hazard ratio: 0.405 – 0.942, which is significantly below 1 (Wald test: df = 1, Chi^2^ = 5.0, p = 0.0254)
