# Supplementary material for: Fitness costs of female choosiness are low in a socially monogamous songbird
Source: PLoS Biol. 2021 Nov 4;19(11):e3001257. doi: 10.1371/journal.pbio.3001257 (PMC8568113; doi:10.1371/journal.pbio.3001257)
Supplement: S1 Text — (DOCX) [file pbio.3001257.s019.docx]

**S1 Text. R-code and model outputs of planned models 1-3 and post-hoc models 4-15.**

**Explanation of variables:**

Dependent variables:

relfit = relative fitness, scaled to a mean of one (12 x number of independent young / total number of independent young in the experimental aviary)

Neggsvalid = Count of genetically verified eggs produced by a female (total sum = 1,080 eggs)

loglatencygen = log10-transformed latency in days to lay the first genetically verified egg

unpaired = not participating in any of the 106 heterosexual pair bonds (0 = no, 1 = yes)

Nsocbonds = number of social pair bonds observed per female (range 0 – 2, total sum = 106)

N_assort_bonds = number of assortative social pair bonds observed per female (range 0 – 2, total sum = 72)

N_disassort_bonds = number of disassortative social pair bonds observed per female (range 0 – 2, total sum = 34)

loglatencysoc = log10-transformed latency in days to the first recorded egg in a clutch attended as one of the 106 social pairs

Singlemomclutches = number of clutches attended as a single mother (range 0 – 2, total sum = 18)

Eggsselfcaredfor = number of eggs for which the female was recorded to attend as a social mother in whatever pairing constellation

Eggsdumpedstrict = number of genetically verified eggs of a female that were cared for by another female

Eggsdumpedwide = number of genetically verified eggs of a female that she did not care for herself (equal to Neggsvalid – Eggsselfcaredfor)

Propdumpedstrict = Relative counts of eggs that a female dumped in the strict sense and her remaining eggs

Propdumpedwide = Relative counts of eggs that a female dumped in the wide sense and her remaining eggs

Propeggsuccess = Relative counts of eggs of a female that developed into independent young and her remaining eggs that did not reach independence

Fixed effects:

trt = female treatment (1 = relaxed competition, 2 = high competition)

Fped = pedigree-based inbreeding coefficient F of the female

Npeers = number of peers reaching independence within the female’s natal aviary

motherfitness = number of independent offspring produced by the mother in the previous year within an equally-long time window

Random effects:

ExpAV = experimental aviary (10 levels)

NatalAV = natal aviary (16 levels)

Ind_ID = female identity (120 levels)

**Code:**

require(lme4)

library("lmerTest")

dd<-read.csv("./choosiness1_females.csv",header=T)

dd$NatalAV<-factor(dd$NatalAV)

dd$ExpAV<-factor(dd$ExpAV)

dd$trt<-factor(dd$trt)

**Model 1a**

m1a<-glm(relfit~trt+scale(Fped,scale=F),data=dd); summary(m1a)

Coefficients:

Estimate Std. Error t value Pr(>|t|)

(Intercept) 0.95523 0.09704 9.844 < 2e-16 ***

trt2 0.06716 0.11884 0.565 0.57308

scale(Fped, scale = F) -3.14271 1.12611 -2.791 0.00614 **

m1aestimates<-glm(relfit~-1+trt+scale(Fped,scale=F),data=dd); summary(m1aestimates)

Coefficients:

Estimate Std. Error t value Pr(>|t|)

trt1 0.95523 0.09704 9.844 < 2e-16 ***

trt2 1.02239 0.06861 14.901 < 2e-16 ***

scale(Fped, scale = F) -3.14271 1.12611 -2.791 0.00614 **

**Model 1b**

m1b<-glm(relfit~trt+scale(Fped,scale=F)+scale(Npeers,scale=F)+scale(motherfitness,scale=F), data=dd); summary(m1b)

Coefficients:

Estimate Std. Error t value Pr(>|t|)

(Intercept) 0.91436 0.10175 8.987 5.94e-15 ***

trt2 0.12846 0.12779 1.005 0.31691

scale(Fped, scale = F) -3.53541 1.25551 -2.816 0.00573 **

scale(Npeers, scale = F) -0.01711 0.01174 -1.457 0.14777

scale(motherfitness, scale = F) 0.03406 0.02049 1.662 0.09925 .

**Model 2**

require(lme4)

m2<-lmer(Neggsvalid~trt+scale(Fped,scale=F)+(1|ExpAV)+(1|NatalAV),data=dd); summary(m2)

Random effects:

Groups Name Variance Std.Dev.

NatalAV (Intercept) 0.1557 0.3945

ExpAV (Intercept) 0.2179 0.4668

Residual 12.4647 3.5305

Number of obs: 120, groups: NatalAV, 15; ExpAV, 10

Fixed effects:

Estimate Std. Error df t value Pr(>|t|)

(Intercept) 8.5633 0.5931 34.7822 14.439 2.94e-16 ***

trt2 0.6400 0.7032 20.1072 0.910 0.37351

scale(Fped, scale = F) -18.8353 6.7343 79.0559 -2.797 0.00648 **

**Model 3**

m3<-lmer(loglatencygen~trt+scale(Fped,scale=F)+(1|ExpAV)+(1|NatalAV),data=dd); summary(m3)

Random effects:

Groups Name Variance Std.Dev.

NatalAV (Intercept) 0.006107 0.07815

ExpAV (Intercept) 0.000000 0.00000

Residual 0.101216 0.31814

Number of obs: 120, groups: NatalAV, 15; ExpAV, 10

Fixed effects:

Estimate Std. Error df t value Pr(>|t|)

(Intercept) 0.92029 0.05662 37.45131 16.255 <2e-16 ***

trt2 -0.03294 0.06884 45.81581 -0.479 0.6346

scale(Fped, scale = F) 1.06877 0.62299 95.07132 1.716 0.0895 .

**Model 4**

m4<-glmer(unpaired~trt+scale(Fped,scale=F)+(1|ExpAV)+(1|NatalAV),data=dd, family=binomial); summary(m4)

Random effects:

Groups Name Variance Std.Dev.

NatalAV (Intercept) 0.03969 0.1992

ExpAV (Intercept) 0.32334 0.5686

Number of obs: 120, groups: NatalAV, 15; ExpAV, 10

Fixed effects:

Estimate Std. Error z value Pr(>|z|)

(Intercept) -2.6337 0.6646 -3.963 7.4e-05 ***

trt2 1.4886 0.7055 2.110 0.03485 *

scale(Fped, scale = F) 20.4028 7.0019 2.914 0.00357 **

# Model 4 Gaussian to obtain approximate df

m4G<-lmer(unpaired~trt+scale(Fped,scale=F)+(1|ExpAV)+(1|NatalAV),data=dd); summary(m4G)

Random effects:

Groups Name Variance Std.Dev.

NatalAV (Intercept) 0.00570 0.07550

ExpAV (Intercept) 0.00408 0.06387

Residual 0.13179 0.36302

Number of obs: 120, groups: NatalAV, 15; ExpAV, 10

Fixed effects:

Estimate Std. Error df t value Pr(>|t|)

(Intercept) 0.09514 0.06596 34.16233 1.442 0.1583

trt2 0.17819 0.07661 9.38302 2.326 0.0439 *

scale(Fped, scale = F) 3.15646 0.71932 95.33756 4.388 2.95e-05 ***

# conservative p-value for treatment

pt(abs(2.11),lower.tail=FALSE,df=9)*2

0.06406977

**Model 5**

m5<-lmer(Nsocbonds~trt+scale(Fped,scale=F)+(1|ExpAV)+(1|NatalAV),data=dd); summary(m5)

Random effects:

Groups Name Variance Std.Dev.

NatalAV (Intercept) 0.014594 0.12081

ExpAV (Intercept) 0.007147 0.08454

Residual 0.247540 0.49753

Number of obs: 120, groups: NatalAV, 15; ExpAV, 10

Fixed effects:

Estimate Std. Error df t value Pr(>|t|)

(Intercept) 0.93126 0.09239 30.78383 10.080 2.86e-11 ***

trt2 -0.08796 0.10762 28.81111 -0.817 0.42042

scale(Fped, scale = F) -3.17993 0.99468 96.68667 -3.197 0.00188 **

**Model 6**

m6<-lmer(N_assort_bonds~trt+scale(Fped,scale=F)+(1|ExpAV)+(1|NatalAV),data=dd); summary(m6)

Random effects:

Groups Name Variance Std.Dev.

NatalAV (Intercept) 0.0000 0.0000

ExpAV (Intercept) 0.0000 0.0000

Residual 0.2212 0.4703

Number of obs: 120, groups: NatalAV, 15; ExpAV, 10

Fixed effects:

Estimate Std. Error df t value Pr(>|t|)

(Intercept) 0.90133 0.07436 117.00000 12.121 < 2e-16 ***

trt2 -0.45200 0.09108 117.00000 -4.963 2.38e-06 ***

scale(Fped, scale = F) -2.26344 0.86299 117.00000 -2.623 0.00988 **

**Model 7**

m7<-lmer(N_disassort_bonds~trt+scale(Fped,scale=F)+(1|ExpAV)+(1|NatalAV),data=dd); summary(m7)

Random effects:

Groups Name Variance Std.Dev.

NatalAV (Intercept) 0.01057 0.1028

ExpAV (Intercept) 0.01089 0.1044

Residual 0.22068 0.4698

Number of obs: 120, groups: NatalAV, 15; ExpAV, 10

Fixed effects:

Estimate Std. Error df t value Pr(>|t|)

(Intercept) 0.03545 0.08836 29.21410 0.401 0.69119

trt2 0.36459 0.09995 21.79147 3.648 0.00144 **

scale(Fped, scale = F) -0.82400 0.94355 97.62335 -0.873 0.38464

**Model 8**

m8<-lmer(loglatencysoc~trt+scale(Fped,scale=F)+(1|ExpAV)+(1|NatalAV),data=dd); summary(m8)

Random effects:

Groups Name Variance Std.Dev.

NatalAV (Intercept) 0.000000 0.00000

ExpAV (Intercept) 0.006349 0.07968

Residual 0.199917 0.44712

Number of obs: 120, groups: NatalAV, 15; ExpAV, 10

Fixed effects:

Estimate Std. Error df t value Pr(>|t|)

(Intercept) 0.89686 0.07505 38.17114 11.950 1.81e-14 ***

trt2 0.23380 0.08659 105.88609 2.700 0.00807 **

scale(Fped, scale = F) 3.65047 0.85214 88.57020 4.284 4.65e-05 ***

**Model 9**

m9<-lmer(Singlemomclutches~trt+scale(Fped,scale=F)+(1|ExpAV)+(1|NatalAV),data=dd); summary(m9)

Random effects:

Groups Name Variance Std.Dev.

NatalAV (Intercept) 0.008803 0.09383

ExpAV (Intercept) 0.000000 0.00000

Residual 0.154795 0.39344

Number of obs: 120, groups: NatalAV, 15; ExpAV, 10

Fixed effects:

Estimate Std. Error df t value Pr(>|t|)

(Intercept) 0.12673 0.06962 33.27238 1.820 0.0777 .

trt2 0.04872 0.08470 40.78098 0.575 0.5684

scale(Fped, scale = F) 1.09214 0.76845 90.67711 1.421 0.1587

**Model 10**

m10<-lmer(Eggsselfcaredfor~trt+scale(Fped,scale=F)+(1|ExpAV)+(1|NatalAV),data=dd); summary(m10)

Random effects:

Groups Name Variance Std.Dev.

NatalAV (Intercept) 0.00 0.000

ExpAV (Intercept) 1.13 1.063

Residual 10.14 3.185

Number of obs: 120, groups: NatalAV, 15; ExpAV, 10

Fixed effects:

Estimate Std. Error df t value Pr(>|t|)

(Intercept) 7.4139 0.6055 28.6811 12.245 6.51e-13 ***

trt2 -0.7834 0.6167 108.0893 -1.270 0.206713

scale(Fped, scale = F) -23.6450 6.3432 111.6922 -3.728 0.000305 ***

**Model 11**

m11<-lmer(Eggsdumpedstrict~trt+scale(Fped,scale=F)+(1|ExpAV)+(1|NatalAV),data=dd); summary(m11)

Random effects:

Groups Name Variance Std.Dev.

NatalAV (Intercept) 0.000 0.000

ExpAV (Intercept) 0.000 0.000

Residual 4.145 2.036

Number of obs: 120, groups: NatalAV, 15; ExpAV, 10

Fixed effects:

Estimate Std. Error df t value Pr(>|t|)

(Intercept) 0.7984 0.3219 117.0000 2.480 0.0146 *

trt2 0.8274 0.3943 117.0000 2.098 0.0380 *

scale(Fped, scale = F) 2.6959 3.7360 117.0000 0.722 0.4720

**Model 12**

m12<-lmer(Eggsdumpedwide~trt+scale(Fped,scale=F)+(1|ExpAV)+(1|NatalAV),data=dd); summary(m12)

Random effects:

Groups Name Variance Std.Dev.

NatalAV (Intercept) 0.2326 0.4823

ExpAV (Intercept) 0.0000 0.0000

Residual 6.9173 2.6301

Number of obs: 120, groups: NatalAV, 15; ExpAV, 10

Fixed effects:

Estimate Std. Error df t value Pr(>|t|)

(Intercept) 1.1143 0.4466 38.9532 2.495 0.01694 *

trt2 1.4819 0.5455 44.0411 2.717 0.00939 **

scale(Fped, scale = F) 3.5141 5.0345 86.4795 0.698 0.48704

**Model 13**

dd$Propdumpedstrict<-cbind(dd$Eggsdumpedstrict,dd$Eggsnotstrictlydumped)

m13<-glmer(Propdumpedstrict~trt+scale(Fped,scale=F)+(1|ExpAV)+(1|NatalAV)+(1|Ind_ID),data=dd, family=binomial); summary(m13)

Random effects:

Groups Name Variance Std.Dev.

Ind_ID (Intercept) 1.969e+00 1.403e+00

NatalAV (Intercept) 2.590e-10 1.609e-05

ExpAV (Intercept) 1.431e-09 3.783e-05

Number of obs: 120, groups: Ind_ID, 120; NatalAV, 15; ExpAV, 10

Fixed effects:

Estimate Std. Error z value Pr(>|z|)

(Intercept) -2.9587 0.3536 -8.367 <2e-16 ***

trt2 0.8687 0.3931 2.210 0.0271 *

scale(Fped, scale = F) 6.7587 3.7493 1.803 0.0714 .

# Model 13 Gaussian to obtain approximate df

dd$Percdumpedstrict=(dd$Eggsdumpedstrict / (dd$Eggsdumpedstrict + dd$Eggsnotstrictlydumped))

m13G<-lmer(Percdumpedstrict~trt+scale(Fped,scale=F)+(1|ExpAV)+(1|NatalAV) ,data=dd); summary(m13G)

Random effects:

Groups Name Variance Std.Dev.

NatalAV (Intercept) 0.00000 0.0000

ExpAV (Intercept) 0.00000 0.0000

Residual 0.04483 0.2117

Number of obs: 116, groups: NatalAV, 15; ExpAV, 10

Fixed effects:

Estimate Std. Error df t value Pr(>|t|)

(Intercept) 0.09053 0.03435 113.00000 2.636 0.00958 **

trt2 0.08311 0.04189 113.00000 1.984 0.04968 *

scale(Fped, scale = F) 0.40447 0.38930 113.00000 1.039 0.30103

# conservative p-value for treatment

pt(abs(2.21),lower.tail=FALSE,df=113)*2

0.02912076

**Model 14**

dd$Propdumpedwide<-cbind(dd$Eggsdumpedwide,dd$Eggsnotwidelydumped)

m14<-glmer(Propdumpedwide~trt+scale(Fped,scale=F)+(1|ExpAV)+(1|NatalAV)+(1|Ind_ID),data=dd, family=binomial); summary(m14)

Random effects:

Groups Name Variance Std.Dev.

Ind_ID (Intercept) 2.38 1.543

NatalAV (Intercept) 0.00 0.000

ExpAV (Intercept) 0.00 0.000

Number of obs: 120, groups: Ind_ID, 120; NatalAV, 15; ExpAV, 10

Fixed effects:

Estimate Std. Error z value Pr(>|z|)

(Intercept) -2.6595 0.3582 -7.425 1.13e-13 ***

trt2 1.3551 0.4065 3.334 0.000857 ***

scale(Fped, scale = F) 12.4515 4.0355 3.085 0.002032 **

# Model 14 Gaussian to obtain approximate df

dd$Percdumpedwide=(dd$Eggsdumpedwide / (dd$Eggsdumpedwide + dd$Eggsnotwidelydumped))

m14G<-lmer(Percdumpedwide~trt+scale(Fped,scale=F)+(1|ExpAV)+(1|NatalAV) ,data=dd); summary(m14G)

Random effects:

Groups Name Variance Std.Dev.

NatalAV (Intercept) 2.231e-03 4.723e-02

ExpAV (Intercept) 2.361e-10 1.537e-05

Residual 6.215e-02 2.493e-01

Number of obs: 116, groups: NatalAV, 15; ExpAV, 10

Fixed effects:

Estimate Std. Error df t value Pr(>|t|)

(Intercept) 0.11870 0.04354 35.03875 2.726 0.009934 **

trt2 0.17326 0.05302 38.76728 3.268 0.002273 **

scale(Fped, scale = F) 1.90790 0.47931 79.17943 3.981 0.000151 ***

# conservative p-value for treatment

pt(abs(3.334),lower.tail=FALSE,df=39)*2

0.001885217

**Model 15**

dd$Propeggsuccess<-cbind(dd$juvd35,dd$Eggsfailed)

m15<-glmer(Propeggsuccess~trt+scale(Fped,scale=F)+(1|ExpAV)+(1|NatalAV)+(1|Ind_ID),data=dd, family=binomial); summary(m15)

Random effects:

Groups Name Variance Std.Dev.

Ind_ID (Intercept) 7.807e-01 8.836e-01

NatalAV (Intercept) 1.758e-10 1.326e-05

ExpAV (Intercept) 7.669e-02 2.769e-01

Number of obs: 120, groups: Ind_ID, 120; NatalAV, 15; ExpAV, 10

Fixed effects:

Estimate Std. Error z value Pr(>|z|)

(Intercept) 0.06413 0.20909 0.307 0.7591

trt2 -0.03649 0.23081 -0.158 0.8744

scale(Fped, scale = F) -6.50954 2.84856 -2.285 0.0223 *

# Model 15 Gaussian to obtain approximate df

dd$Perceggsuccess=(dd$juvd35 / (dd$juvd35 + dd$Eggsfailed))

m15G<-lmer(Perceggsuccess~trt+scale(Fped,scale=F)+(1|ExpAV)+(1|NatalAV) ,data=dd); summary(m15G)

Random effects:

Groups Name Variance Std.Dev.

NatalAV (Intercept) 4.660e-03 6.826e-02

ExpAV (Intercept) 1.963e-11 4.430e-06

Residual 6.565e-02 2.562e-01

Number of obs: 116, groups: NatalAV, 15; ExpAV, 10

Fixed effects:

Estimate Std. Error df t value Pr(>|t|)

(Intercept) 0.50604 0.04743 32.97242 10.669 3.16e-12 ***

trt2 -0.01248 0.05746 40.48904 -0.217 0.829095

scale(Fped, scale = F) -1.72629 0.50707 89.01212 -3.404 0.000996 ***

# conservative p-value for treatment

pt(abs(-0.158),lower.tail=FALSE,df=40)*2

0.8752517

**Model 8a: Cox proportional hazard model**

library(coxme)

library(lmtest)

library(survminer)

dd$latencytofirstbond_event <- 1

dd$latencytofirstbond_event[dd$latencytofirstbond == 75] <- 0

reg1 <- coxme(Surv(latencytofirstbond, latencytofirstbond_event) ~ trt + scale(Fped,scale=F)+(1|ExpAV)+(1|NatalAV), data = dd);summary(reg1)

Cox mixed-effects model fit by maximum likelihood

Data: dd

events, n = 95, 120

Iterations= 16 99

NULL Integrated Fitted

Log-likelihood -399.8088 -391.0575 -388.7493

Chisq df p AIC BIC

Integrated loglik 17.50 4.00 0.00154320 9.50 -0.71

Penalized loglik 22.12 4.07 0.00020329 13.99 3.60

Model: Surv(latencytofirstbond, latencytofirstbond_event) ~ trt + scale(Fped, scale = F) + (1 | ExpAV) + (1 | NatalAV)

Fixed coefficients

coef exp(coef) se(coef) z p

trt2 -0.4819144 0.6175999142 0.2156207 -2.24 0.0250

scale(Fped, scale = F) -8.9509788 0.0001296102 2.8292279 -3.16 0.0016

Random effects

Group Variable Std Dev Variance

ExpAV Intercept 0.182257512 0.033217801

NatalAV Intercept 0.019731828 0.000389345

reg2 <- coxme(Surv(latencytofirstbond, latencytofirstbond_event) ~ scale(Fped,scale=F)+(1|ExpAV)+ (1|NatalAV), data = dd)

waldtest(reg1, reg2, test = "Chisq")

Wald test

Model 1: Surv(latencytofirstbond, latencytofirstbond_event) ~ trt + scale(Fped,

scale = F) + (1 | ExpAV) + (1 | NatalAV)

Model 2: Surv(latencytofirstbond, latencytofirstbond_event) ~ scale(Fped,

scale = F) + (1 | ExpAV) + (1 | NatalAV)

Res.Df Df Chisq Pr(>Chisq)

1 -4.0667

2 -2.7310 -1 4.9953 0.02542 *

exp(coefci(reg1))

2.5 % 97.5 %

trt2 4.047348e-01 0.94241869

scale(Fped, scale = F) 5.062863e-07 0.03318046

plot(survfit(Surv(latencytofirstbond, latencytofirstbond_event) ~ trt, data = dd), lty = c(1,2), ylab="Proportion of single birds", xlab="Time [days]")

lines(survfit(Surv(latencytofirstbond, latencytofirstbond_event) ~ 1, data = dd[dd$trt == 1,]), lty = c(1,2,2), lwd = c(3,1,1), col = "blue" , add = T, mark.time = T)

lines(survfit(Surv(latencytofirstbond, latencytofirstbond_event) ~ 1, data = dd[dd$trt == 2,]), lty = c(1,2,2), lwd = c(3,1,1), col = "red" , add = T, mark.time = T)

legend("topright", c("Relaxed competition", "High competition"), lty = 1, lwd = 2, col = c("blue", "red"), bty = "n", cex =1)

#for output see S1 Fig
