# Supplementary material for: Fitness costs of female choosiness are low in a socially monogamous songbird
Source: PLoS Biol. 2021 Nov 4;19(11):e3001257. doi: 10.1371/journal.pbio.3001257 (PMC8568113; doi:10.1371/journal.pbio.3001257)
Supplement: Attachment — Submitted filename: Reply to reviewers submit.docx [file pbio.3001257.s020.docx]

**Reply to comments on PBIOLOGY-D-21-01052**

**We thank the Senior Editor, the Academic Editor and the three Reviewers for their assessment and**

**constructive suggestions to improve our paper. We addressed all the specific points (in bold) as**

**detailed below.**

**Please note that in this reply, all line information refers to line numbers in the tracked version of our manuscript.**

REVIEWERS' COMMENTS:

Reviewer #1:

[identifies himself as Alexandre Courtiol]

I have read the important study from Forstmeier et al. which empirically explored an understudied aspect of mate choice: the role of mate availability. The paper is well written. The experimental design is simple and elegant. The statistics are suitable. The results are interesting and I salute the authors for having made the effort to pre-register the methods for this study on the Open Science Foundation website. Provided that the authors can successfully address my criticisms, and I am confident they can, I would consider this paper to represent a valuable contribution to the literature of mate choice and, more generally, to the fields of behavioural ecology and evolutionary ecology.

Main remarks:

My main reservation about the paper as it currently stands concerns its overall framing and main result. The authors claim that their experimental setup measures the cost of choosiness and that their results show that the cost is very small if not zero (e.g. lines 256-257). For reasons I will detail next, however, I don't think that the experimental setup actually measures the cost of choosiness, nor that the results show that the cost of choosiness is small. This removes little from the quality and merit of the study, but I think that a different angle should be used to present the exciting results on more solid ground. One option would be to focus on the impact of a change in mate availability.

The literature shows that measuring the cost of choosiness is rarely attempted, and I appreciate that the authors tried to do so. Even if I were willing to accept many practical caveats due to the inherent difficulty of the task, I am not, however, persuaded that the design of this particular study is appropriate for fulfilling this purpose. I will focus on four main issues.

**Reply: We thank the reviewer for these interesting comments, which we have used to refine our Introduction. We now justify our experimental approach more clearly (lines 154-157, 187-200 in the tracked-changes version of our manuscript), explaining why and how we can quantify the cost of choosiness by changing the availability of preferred males. We fully agree with the reviewer that mate availability (relative to the frequency of corresponding preferences) is the key factor in our experiment (lines 31-32). And this key factor is generally thought to cause negative frequency-dependent selection on preferences due to the costs that arise from having preferences that cannot be satisfied (lines 132-149).**

**Overall, it is important to keep in mind that such experimental studies can only be carried out when there are naturally occurring preferences that lead to non-zero choosiness. The consequences of such choosiness can then be explored, and we do this in a naturalistic setting (a relatively complex social environment, lines 155-157). Our experiment enables us to measure the cost of being choosy (with regard to song dialect) under the condition of limited vs practically unlimited availability of preferred mates, reflecting two classical situations that characterize frequency-dependent selection on mate preferences in monogamous species. As we explain below (and in the revised manuscript) we think that our study is close to achieving what can possibly be achieved via experimentation.**

First, I am not convinced by the overall logic of the experimental design: if two treatments are designed so as to influence a cost, then what do we learn about the cost when comparing the two treatments? At best the results directly reflect the artificial design: create very contrasted treatments and you could make the case for a large cost, and create very similar treatments and you could make the case for a small cost.

**Reply: We agree on this conceptual point. If a small measured effect size is the result of a weak treatment, then this would be a rather trivial finding leading to little gain in understanding.**

Here we have two a priori very contrasted treatments but no cost, so what to conclude?

**Reply: We would argue that our finding (of small fitness costs) despite a strong treatment is unexpected (relative to the pre-registration document), which means that we have already learnt something non-trivial.**

Probably that the two treatments did not manipulate the cost as expected, not necessarily that there is no cost.

**Reply: With such an argument, the empirical outcome of our study is used *post hoc* to argue that the treatment must have failed, but this is not a conceptual critique. We clearly disagree with this, also on empirical grounds, because Figures 3, 4, 5B, and 6 illustrate that the treatment had clear consequences, which, however (and interestingly), did not translate into large fitness costs (Figures 2, 5A). From this we conclude that the birds are relatively efficient in maintaining fitness even under unfavourable circumstances (e.g. via egg dumping). This is not a trivial finding of the kind “small effect due to weak treatment”. One could argue that stronger effects on fitness could be provoked with a treatment of varying hetero- versus conspecific mate availability. However, such results would again be perceived as trivial (and failing to address the issue of mate choice among conspecifics).**

In sum, measuring any cost like that tells us a lot about the study design but little about the biology.

**Reply: We respectfully disagree with this conclusion. Our study design matches the general principles of frequency-dependent selection on preferences (lines 132-149), and it reflects important aspects of the biology of socially monogamous species (which we now explain better in the manuscript, lines 117-119, 155-157). Because of this, we argue that we learned something biologically meaningful from this experiment about how behavioural flexibility (e.g. egg dumping) weakens frequency-dependent selection.**

The authors may disagree with me but the general point is that they should justify why they think their experimental design allows for the measurement of the cost of choosiness. It would also be interesting, if they keep their framing as is, to relate the experimental conditions to the extent of the observed variation in mating availability found in nature.

**Reply: We thank the reviewer for making these points, because it helped us improving and clarifying the justification of our study design, explaining why it is biologically relevant for the study species (and perhaps for other socially monogamous species), and for our understanding of mate preferences in general (see lines 31-32, 134-141, 154-157, 187-200).**

Second, the measurement of the cost of choosiness should ideally depend on how choosiness is being defined. Perhaps it should even depend on how one thinks choosiness may have evolved. To give an extreme example, if one considers that cognitive structures have specifically evolved so as to allow individuals to discriminate more precisely between potential mates, then the cost of choosiness should include the cost of growing, maintaining and using such structures; and that would call for a very different type of experimental work than the one presented here. I am not arguing here that the brain is modular, nor that preferences may not have evolved by pure sensory biases, but the previous example illustrates a general problem to which there is an easy fix: the authors should make it very clear what they attempt to measure and what they cannot measure. This is important because that would help the reader to understand what exactly is zero. Probably not the overall cost of choosiness.

**Reply: In the revised Introduction we now explain that we can only experiment with naturally occurring levels of choosiness (e.g. here resulting from song preferences), and we clarify that we do not manipulate choosiness, but rather we examine the consequences of having preferences under two conditions of availability of preferred mates (limited vs not limited). Other hypothetical costs that may arise from the development of specific cognitive abilities are not accessible to experimental research. We are not aware of any study that shows that specific cognitive abilities have evolved for the purpose of mate choice. Nevertheless, we agree that it is worth clarifying what can be addressed with our approach (lines 187-190, 197-200).**

The particular cost the authors aimed at quantifying seems to be related to the decrease in mating rate associated with an increased choosiness. This is not a bad cost to measure and it has indeed been the focus of several theoretical papers, precisely because it is a general cost that may apply across species.

**Reply: We appreciate the reviewer’s comment, because it is interesting to consider how to generalize beyond females of socially monogamous species with bi-parental brood care. However, from our perspective ‘mating rate’ is not a variable that characterizes variation in female reproductive success (for males this variable would be more meaningful).**

Yet, this brings us to the third issue concerning the measurement of choosiness: the measurement of the cost of choosiness should ideally be done under conditions of fixed choosiness and benefits of choice.

**Reply: We agree, but we feel that this is in fact a major strength of our study. As we explain in lines 108-112, 184-185, 195-197, the benefits of choice are kept constant, because song dialect is unrelated to male quality. Thus, females do not gain anything from obtaining a preferred male (relative to a non-preferred male) other than the benefit of the preference being satisfied. Such consequences (“satisfying the preference or not”) are part of the cost of choosiness (see Fig. 1). Regarding ‘fixed choosiness’, females from both treatment groups start with the same strength of preference (for the familiar dialect). During the experiment, females can then adjust their behaviour to the experimentally-induced difference in the availability of the preferred song type. Such flexibility in behaviour is part of the phenomenon we study, because we aimed to quantify the fitness cost of having dialect preferences under two conditions of availability of preferred mates.**

When everything varies, it is not clear how the cost of choosiness can be quantified, even in a model. This is why many models about the evolution of choosiness do not include plasticity. While these conditions of fixed choosiness and benefits are hardly ever met in practice, using metrics such as the number of offspring produced probably makes things worse since these integrative metrics capture, by definition, the whole cost/benefit balance.

**Reply: We find it essential that all beneficial and detrimental consequences of the treatment are getting summarized in terms of relative fitness (lines 154-157, 193-195), because the sum of all fitness effects decides whether choosiness is favoured or selected against (note that in our study we on purpose exclude the benefits of choosiness such that we can measure the costs). Also, it is important that the experimental setup allows the females to behave in a plastic way (lines 155-157). Only this will render the measurement (of the total cost of choosiness) biologically relevant. Hence, while the ‘cost of choosiness’ may be difficult to model (e.g. because one would need decision rules for plastic female behaviour), it is actually easy to quantify empirically, and this should be done at the level of female fitness.**

Perhaps a partial fix would be to also measure the cost using more direct consequences of the choice than the reproductive success, such as something related to the decrease in mating rate (the authors do measure the time to first egg, but why limit this to the first one?). Also, as mentioned above, this point calls for a precise justification about how and why the experimental design allows for the measurement of the cost of choosiness, especially in view of the fact that choosiness and the (perceived) benefits of the choice may vary between treatments.

**Reply: We agree that besides quantifying fitness (Table 1: Model 1) it is also interesting to look at other phenomena (Table 1: Models 2-15) to understand better what happened in the experiment. Mating rate of female zebra finches (e.g. number of copulations per day) is probably not relevant in an evolutionary sense, because it is not (or even negatively) related to female fitness. Sperm can be obtained from almost any male at almost any time. However, in socially monogamous systems with biparental care, females seek the benefits of male parental care, and extra-pair copulations may turn out to be costly in terms of relative fitness if they lead to reduced male help (see lines 115-120). The ‘time to the first (genetic) egg’ was meant to capture the delay in social pairing, but due to frequent egg dumping ‘time to the first egg attended with a social partner’ turned out to be a better measure of the social consequences of the treatment. Regarding the ‘perceived benefits of choice’, this appears to be the same quantity as the ‘cost of unmet preferences’, which we argue to be part of the cost of choosiness (Figure 1).**

Fourth --and this is my biggest reservation--, due to the general difficulty of measuring the cost of choosiness, studying this cost in conditions where it is likely to be high may be easier. The authors seem to agree with this, but I see two inter-related problems with the particular experimental conditions that the authors used. In these conditions, and perhaps in nature, the studied species, Zebra finches, live in high density and are highly promiscuous. It is not clear why anyone would expect an increase in choosiness to trigger a sharp decrease in mating rate in such conditions. In other words, the authors study the cost of choosiness in a species where we already have reasons to believe that it is low. Had they worked on spiders where most individuals die before having had the chance to meet a potential partner, the situation would be quite different. Surprisingly, the authors did seem to expect a high cost of choosiness in their species.

**Reply: We have modified the Introduction to better explain the mating system of zebra finches and the factors that are likely to be important for determining female fitness. Mating rate is not a limiting factor for female fitness. Females primarily seek benefits from male parental care, and genetic monogamy may be essential for productive, long-term partnerships. Our aim is to capture all consequences of the treatment in terms of effects on relative fitness (lines 154-157, 193-200). We expected that ‘remaining unpaired’ would have large fitness consequences (which turned out to be smaller due to egg dumping), and ‘pairing disassortatively’ could have led to “dysfunctional relationships” with high levels of extra-pair paternity and low levels of male care and hence lower fitness (but it did not). Note that such “dysfunctional relationships” had been observed previously (Ihle et al. 2015) with a treatment that brakes up behavioural compatibility between partners, so our expectation was reasonable. The spider example seems so extreme that we doubt that high levels of choosiness (rejection of conspecifics) could have evolved under such circumstances. Yet in light of this example we have modified our statement about costs of choosiness being highest in monogamous mating systems (line 28)**

In fact, the key premise of the study is that "Selection against choosiness should be strongest in socially monogamous mating systems" (from the abstract) because the mating system is shaping the costs of choosiness. I may agree with that if they would have dropped the word "socially", but that word is important. Indeed, if selection is measured via genetic contributions, then the costs and benefits of choosiness should be shaped by the genetic mating system, irrespective of the social one. Of course, whether birds do find a social mate does impact on their realised fitness. But it does so not through shaping the costs and benefits of choosiness for genetic mates; it does so through shaping the costs and benefits of choosiness for social mates. In sum, I see a discrepancy between the level at which the experimental design plays out (social level) and the level at which the cost of choosiness is being measured (number of eggs, i.e. genetic level). Due to this discrepancy, I fear that the study cannot

actually "quantify the fitness costs of having mating preferences that are difficult to satisfy" (from the abstract), since indeed as the authors show, the preferences are not particularly difficult to satisfy at the genetic level.

**Reply: Regarding the dichotomy between the social and the genetic mating system, we argue that the full fitness consequences can only be measured in a meaningful way when the social and genetic mating systems are affecting each other, as they would be in nature. Social mate choice and extra-pair mate choice take place in a complex social environment, and the consequences of choosiness (of preferring the familiar dialect) need to be examined in this complicated setting (including the consequences of social mates potentially reducing parental care in response to extra-pair copulations by their partner). Hence, preferences may be ‘satisfied at the genetic level’ but the indirect consequences for fitness need to be assessed within a naturalistic setting (where social mates can react to their partner’s extra-pair behaviour, lines 115-120). Note that it is not certain whether cuckolded males reduce their parental care (as this is rather difficult to prove) or tend to divorce their partners, but for our study it only matters that such consequences are captured by the measure of female fitness.**

Perhaps this is a matter of semantics, but I think that there is more to it than that. The authors seem to regret that the theoretical literature disregards what females may do when their preferences are not satisfied. For instance, the authors go on to propose that females may opt for extra pair copulations. However, from the theoretical perspective of the models discussed (at least for the ones I know), there is no issue: if females can satisfy their preferences --whether directly, or via extra pair copulations-- then their preferences are precisely satisfied, and the question of what they do when they are not becomes moot.

**Reply: Satisfaction of preferences at the genetic level will have no fitness consequences in our setup (genetic quality is the same for both dialects). We argue that it is important to study all consequences of not being able to obtain a preferred social mate and of engaging in extra-pair mating in terms of relative fitness (including any “punishment” by the partner, lines 154-157, 193-195).**

To suggest a solution to what I have described, I propose to frame the study as investigating the impact of mate availability (which is indeed one important aspect related to choosiness). This would avoid falling into HARKing while still doing justice to the pre-registered methodology. I do want to emphasize that I think that the design is great and reveals very interesting information about the biology of mate choice. I just don't think that it measures the cost of choosiness in any meaningful way. I also vaguely thought of perhaps trying to decompose the cost further (i.e. disentangling the cost of choice for the social partner from that of the choice for the genetic partner), but the authors would know best what to do.

**Reply: Our Introduction clarifies how we use manipulation of the availability of preferred males to study the fitness consequences of having such a preference. Females from both treatment groups have such preferences (they are both choosy) but the consequences depend on availability, which lies at the heart of frequency-dependent selection of preferences (i.e. costs of wanting the same limited resource most others want). See lines 31-32, 187-193.**

Do my criticisms imply that this study is weak? No. Not at all. This study reveals an interesting relationship between social and genetic pairings: when preferences are easy to satisfy socially, social and genetic pairings seem very much in line. When they are not, then the correlation between social and genetic pairing decreases, with the social choice becoming more random and the genetic choice remaining unaltered. I don't know the bird literature well enough to know if this is already well known and established, but I find this very interesting. Indeed, this result shows, at least in zebra finches and under these experimental conditions, that what matters is fulfilling one's preferences for song dialects at the genetic level. This result may give insights about the benefits related to the particular preferences for the trait. The decrease in the correlation between social and genetic pairings also shows that individuals are capable of plasticity that seems adaptive.

Whether or not this reflects the result of past selection in the wild is an interesting question. One could speculate that such plasticity would have been selected for the particular reason that the correlation between genetic preferences and social mate availability varies substantially. Or perhaps, this reflects plasticity that would have evolved for more general purposes… It also begs the question of why resistance against brood parasitism has not evolved. I vaguely remember works from Bielefeld people or their colleagues that did show that kin recognition matters for parental care after hatching, perhaps this would be a counter strategy. Or perhaps tolerance is a kind of cooperation that would make sense in the natural conditions.

**Reply: We thank the reviewer for these interesting thoughts. We agree that the question remains how this would play out in the wild. Please, note that we are sceptical regarding claims of kin discrimination, as the overall evidence seems weak (see also the high fitness of egg-dumping females). However, if kin discrimination indeed occurs, its consequences in terms of relative fitness would be fully covered in our study.**

In short, this study is very interesting, well done, and thought provoking. A small rephrasing of the introduction and the discussion would suffice to avoid all the aforementioned problems and turn this work into a great paper for Plos Biology.

**Reply: We appreciate the overall positive appraisal of our study and the interesting and thought-provoking comments above. We have rephrased parts of the Introduction to clarify the rationale of the study (see lines 154-157, 186-200).**

Minor general remarks:

One interesting set of figures that could be added to the paper (or as SI) would be a comparison between social and genetic levels. In particular, one figure could represent the number/proportion of assortative and disassortative pairings within each experimental treatment comparing both for social and genetic partners. Unless I missed it, in the current presentation one cannot directly tell how strong the assortative mating is at the genetic level in each treatment. (The pooled numbers are given line 190 and social level in figure 4).

**Reply: We now provide these interesting numbers (proportion of genetic eggs that are assortative within each of the two treatments) in lines 250-251. We think that all other information is presented in Figures 4 and 6.**

I would also find it interesting to see the breakdown of relative fitness and egg dumping in this light: genetic vs social for each treatment. That is, to make the equivalent of figure 5 for the genetic level. That way it would be easier for the reader to directly compare the social and genetic levels throughout.

**Reply: We now clarify that Figure 5 shows data at the genetic level from the female perspective (line 862). We are not certain how these numbers could be further broken down in a way that would be related to the topic of our study.**

Statistics: the statistics are generally very good. I only have 2 small remarks:

The authors sometimes seem to switch from (G)LMMs to simple t-tests (e.g. line 227). It is not clear to me why they do so. I don't think they have justified it. If the switch is to account for heteroskedasticity, the authors may want to try using the R package spaMM, which would allow them to do everything lme4 can do, but also much more, including defining a model for the residual variance. The syntax would thus resemble something like: spaMM::fitme(response ~ trt + ... + (1|ExpAV) + (1|NatalAV), resid.model = ~ trt, data = dd, family = ...). The R package glmmTMB and its dispformula argument would be another alternative. I am not sure this is required here but spaMM would also allow one to define random effects acting on the residual variance (i.e. spaMM can fit DHGLM sensu Lee & Nelder).

I think authors could do a little better than "p-values were calculated from t-values assuming infinite degrees of freedom" (line 437), which is not a conservative approach. Perhaps the authors did not do that anyhow since I see corrected degrees of freedom in the tables. Yet, if anything the degrees of freedom should be approximated, it would probably be best to underestimate them instead of overestimating them since an increase in degrees of freedom leads to more false positives. Anyhow, there is no need to reinvent the wheel since it has become straightforward these days to test the effect of variables in (G)LMM using parametric bootstrap (or applying a Kenward-Roger approximation, although I don't recall if that latter approach is appropriate for GLMM too). This is true irrespective of using lme4, spaMM or glmmTMB since some of these packages directly offer these options (e.g. spaMM), or have companion packages developed for such purpose (e.g. the R package pbkrtest

handles lme4 fits, and others exist too).

**Reply: We appreciate the many constructive suggestions made by all three reviewers on this topic. After exploring a range of options, we have tried to implement the most pragmatic solution for each problem at hand, and to present alternative ways of analysis only as Supplementary material (e.g. Cox model suggested by Reviewer 2). Please bear in mind that the multitude of options of analysis creates a dilemma of how to avoid selective reporting (e.g. of the method that yields the lowest p-values). We therefore try to adhere to the pre-registered methods (for models 1-3) as much as possible when carrying out the exploratory data analysis (models 4-15). We explored the suggested option of parametric bootstrapping (using the lmeresampler package) and obtained considerably lower p-values for instance for the important model 4. We are not sure why, but we are worried that the resampling procedure might be inadequate for the given experimental design, hence we did not include this in the Supplement.**

**Regarding the first issue on t-tests, we now explain (lines 599-600) that we use t-tests for exploring (post hoc) specific subsets of the data. We think this is particularly useful for making a clear distinction between the structured tests for treatment effects per se (all listed in Table 1) and data exploration on subsets of data that are not under experimental control (e.g. to describe the behaviour of all unpaired or assortatively paired females).**

**Regarding the second issue of degrees of freedom, please note that line 437 (as quoted above) should have referred to z-values (not t-values), which was our mistake. The lmerTest package that we used, uses the indicated Satterthwaite degrees of freedom to calculate p from t (in all glm and lmer models), and only in the case of glmer models (models 4, 13, 14, and 15) p is calculated from z assuming infinite dfs. To fix this issue (of anti-conservative p-values) in the most pragmatic way (following Reviewer 3), we refitted those models as Gaussian models, obtained the estimated Satterthwaite df, and then calculated a more conservative p-value from the original z-value using these reduced dfs (see lines 538-543). We decided to use these more conservative p-values throughout (see Table 1), even though we obtained less-conservative results when implementing parametric bootstrapping.**

Line-by-line remarks:

Line 59-61: I would appreciate it if the authors would illustrate their statement with a few names of species where indeed most or all females mate with the same lekking male.

**Reply: We added two extreme examples to line 66.**

Line 71: my reading of these papers is that the indirect benefits are small but not that the costs are small; if the authors agree with me, they should rephrase their statement.

**Reply: We rephrased the statement (line 77), but would like to note that the costs have hardly ever been measured on the relevant scale of female fitness, and that costs in other currencies (like time) may have been overinterpreted.**

Line 100: "As a consequence, such preferences will be strongly selected against" -> "can be strongly selected against". Indeed, it all depends on the difference in reproductive success between females that did meet their preferences and those that did not. If the difference is large enough, it won't be selected against. Which is why the authors correctly continued with "particularly when a male ornament is a poor indicator...".

**Reply: Changed as suggested to “can be” (line 144)**

Lines 136-137: it is not clear in this paragraph if the 4 vs 8 / 8 vs 4 is just an example or the only two experimental conditions used in the study. We later learn the latter interpretation is correct.

**Reply: We now make this explicit at this point of the manuscript (see line 176)**

Line 158: it is indicated that 556 offsprings reached independence and figure 3 indicates that 1074 eggs were fertilized. The authors should mention and explain the large discrepancy between these 2 numbers.

**Reply: We now explain (line 298-299) that the discrepancy results from embryo and nestling mortality which is usually so high in these populations.**

Line 169 and elsewhere: the authors indicate an interquartile range but the distribution on which such range applies is not specified. Is that the interquartile range of raw data? Of the back-transformed means? Of something else?

**Reply: We clarified that this is on the raw data (line 223)**

Line 173: I would not write "reduce" here but instead speak of cost avoidance.

**Reply: Changed as suggested (line 227)**

Lines 187-188: the authors should perhaps clarify what the maximum achievable number of assortative pairs is (80 if I got it right, which does illustrate that 72 is very close to expectations under perfect assortative mating).

**Reply: This interpretation is not quite accurate. To clarify this issue, we now added a description of the social bonds in the Methods section (line 514-517). Please note that our definition of a heterosexual pair attending a clutch together does not exclude the possibility of social polygamy or re-pairing. Hence, the 106 social pairs involved only 95 different males and 95 different females (added to line 245). Note that this is also visible from Figure 4A and B, where some individuals engage in multiple bonds (see the “mixed” category).**

Line 208: authors should add the degrees of freedom for the results cited that are not in a table.

**Reply: Changed accordingly (line 271)**

Line 215: authors should recall the p-value of the non-significant results, as they did for significant ones.

**Reply: Changed accordingly (line 281, 298)**

Lines 211-223: authors could try to provide effect sizes in the text or illustration as figures for readers to get a sense of how different females from both groups behave without having to dig into SI tables.

**Reply: Note that the respective values are all presented in Table 1, which will be displayed next to this text, and the corresponding Supplementary Tables are only needed if readers want to check the details of the underlying model. We therefore prefer to avoid repeating the information presented in Table 1 in the text.**

Lines 256-257 + 301-302: I disagree (see main comments above).

**Reply: We hope that the arguments above and the revised Introduction and Discussion help clarifying why we think these conclusions are justified.**

Line 307: indeed it would be good if the authors discuss the differences between the results they show here and those they published in ref 25. One important aspect is how the benefits of choice are being influenced in the two studies.

**Reply: We added a sentence (line 391-394) explaining that mate choice for behavioural compatibility appears to be important in this species. In the current study some pairs were mismatched for song (apparently with little detrimental effects on fitness), but the birds still had the opportunity to freely choose a partner. The earlier study [25], in contrast, used forced pairings (i.e. it was designed to break up behavioural compatibility), and found larger effects on fitness.**

Figure 1: the y-axis should not be labelled "relative female fitness", since, by definition, the mean relative female fitness should always be 1. I would perhaps remove the 1, keep the 0 and label the axis as "female fitness".

**Reply: Changed accordingly**

Figure 2: perhaps the authors should indicate some more information for the non r-iens: that data have been jittered horizontally for visual purposes, that those are boxplots, what whiskers represent… They did however mention that the horizontal bars indicate means rather than the (default) median, which is the most important detail since everything else is quite standard. (Same for fig 5).

**Reply: Changed accordingly (line 827-830, 866). Note that we had stated incorrectly that the graph shows means rather than medians (default setting) since means and medians happened to be identical to the nearest percent.**

Figure 4: the authors may want to revise their choice of colour so that B&W printing works too.

**Reply: Changed accordingly**

Alexandre Courtiol

Reviewer #2:

[identifies himself as Pr François-Xavier Dechaume-Moncharmont]

This manuscript deals with an important and largely underexplored question, the actual fitness cost of choosiness during pair formation. The experimental study is well designed using a very large dataset (8 cultural lines, 120 focal females, 289 clutches, 1399 eggs, 556 offspring over 4 months experiments). These results are highly valuable, and they will surely interest large audience readership. I also generally appreciated the thorough statistical analyses and the clarity of the methodological description about these questions in the Material and Methods section. The authors are fully transparent about the a priori and post-hoc hypotheses (through pre-registered protocol). They took great care to confirm genetics maternity and to consider inbreeding in the analyses. My overall impression about this manuscript is thus largely positive. I recommend acceptation after revisions because some points still deserve clarification in the methods, analysis and discussion.

Major comments

A major limitation of the present is that male traits submitted to female preference is a song dialect. The authors have extended and recognized expertise on this question: in previous works and in the present MS (preliminary tests), they provided convincing evidence that there is assortative pairing for dialect in this species. They also prudently try to explain the adaptive significance of such preference in the discussion (Lines 283 and followings). Yet, the crucial points here is that this trait is almost costless for the male, and is not genetically inherited (the song repertoire has been learned by imitation during ontogeny). Therefore, it does not fully satisfy two important components of sexual selection. (1) Honest signal: it makes sense for the female to choose a male based on a given traits if this trait is correlated with either the fitness quality of the male or the (direct or indirect) fitness gain for the female. (2) Heritability: to allow co-evolution between

signal in males and preference in females, the male trait should be heritable. In the present study, the heritability of the male dialect is extremely weak, and only by cultural transmission (as nicely illustrated by the cross-fostering design in preliminary experiments), and the correlation between male trait and potential male quality is weakly discussed, and only as possible explanation in the discussion. This could be an issue as this study investigates the fitness gain arising from partner choice in absence of competition, or its symmetric, the fitness cost arising from absence of choice under competition. If the male's dialect does not indicate its quality, one could not expect large effects of female choice on her fitness. I recommend that the authors address this possible limitation more explicitly and earlier in the MS.

**Reply: We now explain more clearly (line 108-112, 184-186, 195-200 in the tracked-changes version of our manuscript) that** **for our goal of quantifying the costs of choosiness it is essential to be able to separate the benefits of choice from the costs of being choosy. This separation is achieved by studying preferences for a trait (familiar song dialect) that does not convey any fitness benefits to the female per se (male quality is kept constant). Thus, any effect on the female of having a preference satisfied (or not) is part of the costs of choosiness (Figure 1) and cannot be ascribed to variation in male quality. Although we understand that preferences for honest and heritable signals might be somewhat more interesting than preferences for familiar dialects, it is only the latter that allows us to quantify the costs of choosiness in the absence of variation in benefits. Also note that in 20 years of research on zebra finch mate choice, we have not been able to detect any preferences for honest and heritable signals of male quality.**

Minor comments

Lines 101, 109 and 138. The authors refer to a game theory process of "negative frequency dependence". The associated references (34-37) do not help to see why they use this concept here. The definition given lines 101-103 is incorrect. "Negative frequency dependence" refers to situation in which rare morphs (or strategies) are favoured compared to more frequent morphs. I do not understand its relevance in the cases discussed in the present MS. Clarification or rewriting are requested here.

**Reply: We have now revised this section, expanding our explanation (line 132-149)** **why female mate preferences in socially monogamous species are subject to frequency-dependent selection. Lines 101-103 of the former version were not meant to provide a definition (now given in lines 134-135), and the listed references were about studies that had previously stated that preferences are subject to frequency-dependent selection (though this is not always visible from the title or abstract).**

While I acknowledge the attempt of clarification of the several fitness costs of choosiness in females (Fig. 1), I was highly disturbed by the wording "cost of dissatisfaction" because this arrow can also represent the cost due to poor male quality. This arrow aggregates both opportunity costs for the female and costs due to bad male quality. Would it be possible to use a less anthropomorphic wording choice?

**Reply: As explained above (and in lines 107-120, 195-200, and the legend of Fig. 1),** **our study allows to separate benefits of choice from costs of choosiness, because males of either dialect have the same average quality. The only effect in this respect that remains, is a “psychological” effect on the female in terms of having her preference satisfied or not. We have revised the wording accordingly (“cost of unmet preferences”, see Fig. 1) to be clear and not risking to sound anthropomorphic.**

Statistical analysis. Several models used scaling of covariates to report effect size measures (for instance line 711), but this scaling only subtracted the mean and did not divide by the standard deviation as illustrated by the corresponding R code line 812 in the parameter of the function scale(…, scale = FALSE). Could the authors justify this choice? If they aim at providing standardized effect sizes to allow comparisons (within or between studies), I consider that full scaling (mean and variance) is more appropriate.

**Reply: We now explain (lines 551-557) that the purpose of this mean-centering of covariates is to render the intercept and the treatment contrast directly interpretable (i.e. they should approximately match the means of the two treatment groups; see Schielzeth 2010).**

Relative fitness gain. I was also disturbed by the calculation of relative fitness gain. While the provided equation (line 422) centres the average value on 1 for each aviary, it does not control for unequal variance between aviary. I consider that relative fitness gain for female i should be calculated as scaled metrics : RF_i = (F_i - mu)/sd, were F_i was the actual fitness of female i, mu is the mean fitness of the female in the aviary and sd the standard deviation of the fitness in the aviary. Using this scaling transformation, the mean RF in each aviary is the same (equal to zero) and the variance is equal to 1. The authors should justify their calculation choice focusing only on mean scaling and not mean and variance scaling.

**Reply: The measure of relative fitness (as we use it) is firmly established in evolutionary theory and quantitative genetics, as it directly relates to the relative number of offspring contributed to the next generation (**<https://www.biologyonline.com/dictionary/relative-fitness>**). Although a range of standardizations can be found depending on the purpose (Orr 2009 Nat Rev Genet 10:531-539), our measure is most useful for the present goal. The measure ensures comparability between aviaries and should not be adjusted for idiosyncratic variability in fitness between aviaries. For instance, a relative fitness of 0.5 indicates that the female produces only half the number of offspring compared to the population mean, and this interpretation is the same for each aviary. This is why we pre-registered this metric of relative fitness as the primary outcome measure of our experiment. In contrast, the suggested z-scaling, would mean that such females (of relative fitness = 0.5) would get ascribed varying values (e.g. -1.0, -0.5, -1.5) depending on how much variance in fitness there was in each aviary, despite their relative contributions to their populations being identical.**

Lines 202 and followings. There is a far better way to cope with censored data (when the female did not lay eggs) than exclude them from the analysis. Cox proportional hazard model are design for that very purpose, with time data. Instead of dumping the information that a female has not yet lay egg, it fully uses the information that no eggs have been laid at the end of the observation period. These models are easy to implement in R and their analyses are straightforward. I recommend that the authors analyse all times latency data as censored variables, bounded by the end of the experimental period using Cox models.

**Reply: We now support the results of model 8 by showing the corresponding Cox model (model 8a, S17 Table, S1 Figure). We present this as an additional follow-up test (rather than replacing model 8 with model 8a) in order to maintain full transparency about all the analyses that were conducted (i.e. model 8 remains in Table 1). The same could be done for the pre-registered model 3, but we felt that this was not necessary given that the treatment effect in model 3 is clearly non-significant, and that only 3 out of 120 females had been assigned a minimum latency of 75 days as they did not lay any eggs (see lines 271-274, 592-598, 888, 906, 942, 949).**

Figure 4 A and B, such pie charts are poorly informative and should be avoided. It's easy to replace them by two quick sentences in the main text.

**Reply: We know that pie charts have been criticised for being simplistic, but there is also something to say for the idea that important findings of a study (see Abstract) should be illustrated visually. Please note that these pie charts are intuitive to read, and they present a total of 16 numbers (proportions and sample sizes) which would be relatively much to pack into one or two sentences. Moreover, the illustration helps with defining the four categories of pairing which is subsequently used in Figure 5 (which refers back to Figure 4A, B). Hence, we felt that it was not practical to take out these pie charts from Figure 4 (and Figure 4C, D cannot easily be used for defining four categories).**

Line 292. The reference is missing in the reference list.

**Reply: Fixed accordingly (see line 378)**

--

Pr François-Xavier Dechaume-Moncharmont

University of Lyon, France

[fx.dechaume@univ-lyon1.fr](mailto:fx.dechaume@univ-lyon1.fr)

Reviewer #3:

This study by Forstmeier et al. estimates the costs of female choosiness in the context of mate choice in captive populations of zebra finches. Estimating the costs of female preferences is important because such costs play a large role in determining expected outcomes in models of sexual selection by mate choice. I was quite impressed by this study, which is based on an imaginative design that exploits the finches' preference for 'local' (i.e. natal) song dialects. A clever cross-fostering design was also employed to avoid many potential statistical issues. The study was based on a pre-registered protocol. The authors did not find the result they expected (females that could not meet their preferences did not suffer an overall fitness cost) and they consequently also include exploratory (non-registered) analyses designed to uncover the reasons for this surprising 'non-result'. I appreciated the authors' transparency concerning the discrepancies between the pre-registered

analyses and the current study and think the deviations are entirely reasonable and justified by the data. Their major unexpected finding was that females who remained unpaired (presumably due to the lack of preferred males) made up for this by dumping eggs in other birds' nests.

I have little in the way of major comments, as I found this article rigorous and well-written.

1. Socially monogamous and lekking systems differs not only in the costs of female choose (potentially higher under monogamy) but also potentially in the benefits (e.g. if there is large variation in direct benefits such as parental care provided by males). In my view, this could have explained more clearly in the introduction.

**Reply: We tried to explain this more clearly (lines 84-90 in the tracked-changes version of our manuscript.)**

2. In the discussion, I believe it would be helpful to speculate on the similarities/differences between aviary and wild conditions for this species. E.g. I'm aware that zebra finches often nest colonially in the wild - is the opportunity for egg dumping similar or much higher under aviary conditions? This is relevant to estimating the costs of choice during the species' evolutionary history (especially for the 'recently wild' lines).

**Reply: We appreciate the point made by the reviewer. We added information on the rates of egg dumping in a range of captive and wild settings, which further strengthen the conclusion that rates of egg dumping were markedly higher in the high-competition females than previously reported (lines 345-350).**

3. The authors assumed infinite degrees of freedom for the t-tests based on mixed models. I'm aware that obtaining p-values from mixed models is a controversial topic and I am by no means an expert. My understanding, however, is that it is unwarranted (and anti-conservative) to assume infinite df in cases like this with a relatively small number of groups. I would suggest trying to roughly estimate the degrees of freedom based on similar fixed designs or using another approach (e.g. parametric bootstrap). If the original assumption is retained, it should be justified more rigorously.

**Reply: We now estimate degrees of freedom from the corresponding Gaussian mixed effect models, and then manually calculate a more conservative p-value from the z-value of our binomial models #4, 13, 14, and 15 (see lines 538-543 and the corresponding Supplementary Tables).**

4. One potential experimental flaw is already revealed (with refreshing transparency, I might add) by the authors:

This allocation procedure may have introduced a bias, because rearing aviaries that were highly productive (had more offspring) were more frequently designated to send groups of 8 offspring to an experimental aviary, while those that produced fewer offspring (in the extreme case fewer than 8 of one sex) were more likely used to send a group of 4 offspring to an experimental aviary. This might bias our fitness estimates if offspring production was partly heritable or if housing density prior to the experiment influenced the fitness in the experimental aviaries.

I think the authors did a good enough job of accounting for this potential source of error. However, I think they should refer to it in the discussion as well as the methods.

**Reply: We now get back to this point in the Discussion (see lines 326-330)**

Otherwise I have only have minor comments:

L28: 'Selection against choosiness should be strongest in socially monogamous mating systems': This statement only applies to the choice of social mate, not to extra-pair mates.

**Reply: We hope that this point will be sufficiently clear in the revised Introduction. In the Abstract there is little space for further explanations. Note that we slightly toned down the statement in the Abstract in light of comments by Reviewer 1 (line 28).**

L52: I would say 'fixed optimal level', as plastic responses can also be optimal

**Reply: Changed accordingly (line 57)**

L54-5: Please add some citations to the huge theoretical literature on this topic. E.g.

Iwasa & Pomiankowski (1999). Good Parent and Good Genes Models of Handicap Evolution. (doi: 10.1006/jtbi.1999.0979)

Kokko et al. (2015). Mate-sampling costs and sexy sons. (10.1111/jeb.12532)

**Reply: Changed accordingly (line 60)**

L59: 'The most spectacular examples of sexually selected traits have been observed in lek mating systems with strong reproductive skew'. The word 'spectacular' is subjective, of course, but I would not make this claim. Many paradigmatic examples (e.g. some birds-of-paradise) are not lekking species. 'Many of the most' would be better in my view.

**Reply: Changed to “Some of the most spectacular examples…” (line 64)**

L74: I would write 'more tractable' rather than 'better'. Monogamy with direct benefits is quite a different scenario from a theoretical perspective and the questions answered by studying monogamous and lek systems are not necessarily the same

**Reply: Changed accordingly (line 80)**

L94-99: Another possible cost arises if non-preferred partners provide lower direct benefits (e.g. are worse parents). Also, this sentence is very difficult to read - I suggest rephrasing.

**Reply: Sentence was rephrased to be more easily understandable and to also clarify the role of variation in partner quality (lines 107-120)**

L105: This point is made very clearly by Fitzpatrick and Servedio (2018, doi: 10.1093/cz/zoy029) in the context of male mate choice.

**Reply: Reference added (line 132)**

L113: 'relatively little consensus': or simply a lack of strong preferences

**Reply: Changed accordingly (line 147-148)**

L224-228: It's not clear which comparisons these t-tests represent.

**Reply: Now clarified (line 292-294)**

L283: 'assortative mating was present … at the genetic level': I puzzled over this for a while, because song dialect is not genetically determined. I now realize you meant to contrast genetic paternity with social pairing. I suggest rephrasing this so that it's easier to digest.

**Reply: Rephrased to make clear (line 362-364)**

L297-298: 'whereby we predicted that these costs' -> 'which we predicted'

**Reply: Changed accordingly (line 379)**

L299: 'Our study did not fail in the sense that our treatment was effective': I suggest framing more positively, e.g. 'Although our experimental treatment was effective in eliciting strong assortative mating preferences'

**Reply: Changed accordingly (line 380-381)**

Typos etc.

**Reply: All changed accordingly (lines 44, 295, 106, 181, 208, 315, 379, 387, 394, 448, 490)**

L43: 'equal fitness as' -> 'equal fitness to'. Similarly on L229.

L95: delete comma before 'can'

L141: 'two-third majority' -> 'two-thirds majority'

L154: delete 'part of' (it reads weird and it's clear that only part of the analysis was unplanned)

L246: 'inform us about'. Other alternatives (perhaps preferable): 'inform discussions about' or 'help frame discussions about'

L305: delete comma before 'were'

L309: 'models on' -> 'models of'

L353: Does the 'Hence' belong here?

L393: 'naturally died embryos and nestlings' -> 'embryos and nestlings that died naturally' [or 'of natural causes']

L393: 'naturally died' -> 'dead'? Or rephrase as above.

Fig. 3: get rid of the red squiggles from Word

Fig. 5 legend: 'wide' -> 'broad' for consistency

**Reply: Note that “wide” had been used throughout the document (20 times including Supplement) while “broad” was never used.**
