# Supplementary material for: Fitness costs of female choosiness are low in a socially monogamous songbird
Source: PLoS Biol. 2021 Nov 4;19(11):e3001257. doi: 10.1371/journal.pbio.3001257 (PMC8568113; doi:10.1371/journal.pbio.3001257)
Supplement: Attachment — Submitted filename: Reply to Editor Comments.docx [file pbio.3001257.s021.docx]

**Reply to Editor Comments on PBIOLOGY-D-21-01052_R1**

IMPORTANT:

a) We wonder if you could provide a more informative and declarative title. We usually prefer titles that contain an active verb and which contain no punctuation. Something along the lines of "Socially monogamous females avoid costs of choosiness by XYZ..." or "Female choosiness in a socially monogamous bird incurs XYZ costs...." might do, but I do realise that the findings are complex. I'm happy to discuss this further if you like.

**Reply: We agree that the title should declare what we found. The pre-registered goal of our study was to quantify the fitness costs of choosiness. The previous title was “Fitness costs of female choosiness in a socially monogamous songbird”, and we have now changed this to “Low fitness costs of female choosiness in a socially monogamous songbird”. Alternatively, if you prefer an active verb, we could also use “Fitness costs of female choosiness are low in a socially monogamous songbird”. These two versions are our preferred options. More complex titles that focus on how the females overcome the costs of choosiness (e.g. via egg dumping) would somewhat distract from the original goal of our study and would focus on post-hoc data interpretation, which we would find unfortunate for a pre-registered study. Instead, the result of the quantification should remain in the focus, especially given that no previous study has carried out such a quantification before.**

b) Many thanks for providing the underlying data in OSF. Please could you indicate the location of these data clearly in each relevant main and supplementary Figure legend? e.g. "The data underlying this Figure may be found in <https://osf.io/6e8np/>"

**Reply: We have added this statement to all Figure Legends (except for the conceptual Fig. 1) including the Supplementary S1 Figure. We have edited the Captions such that they now consist of Label, Title, and Legend, and we have moved them to the place in the text where the Figure should be inserted. The Figures we now provide as separate TIFF files using the PACE tool.**

As you address these items, please take this last chance to review your reference list to ensure that it is complete and correct. If you have cited papers that have been retracted, please include the rationale for doing so in the manuscript text, or remove these references and replace them with relevant current references. Any changes to the reference list should be mentioned in the cover letter that accompanies your revised manuscript.

**Reply: We have checked all references accordingly.**

**In addition, we have used this final round of revision to make some minor edits on the text in order to improve the English. In the file “Costs of choosiness MSonly_R2_tracked_submit.docx” all changes are highlighted using the ‘Track Changes’ function of Word. We hope that you agree with these edits.**
